# Supplementary material for: Probing a neural unreliability account of auditory sensory processing atypicalities in Rett Syndrome
Source: J Neurodev Disord. 2024 Jun 3;16:28. doi: 10.1186/s11689-024-09544-x (PMC11149250; doi:10.1186/s11689-024-09544-x)
Supplement: Supplementary file 1 — Supplementary Material file 1. [file 11689_2024_9544_MOESM1_ESM.docx]

**Supplementary figures:**


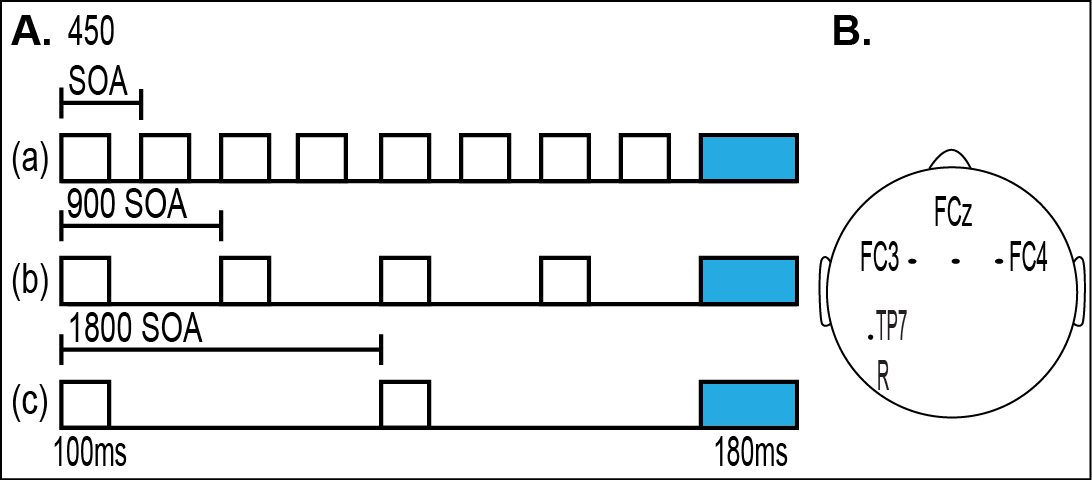
.

**Figure S1:** Oddball paradigm design: A. 3 experimental conditions with varied stimulus presentation intervals (SOA): (a) 450 ms intervals between stimuli, (b) 900 ms between stimuli and (c) 1800 ms between stimuli. B. Shows a map of the electrode site of interest (FC3, FCz, and FC4) and reference electrode R (TP7).

| **Subject** | **Age** | **Mutations** | **(RSSS)** | **Seizures** | **Seizure OS** | **Ambulatory** | **Medications** | **Age of R** |
| --- | --- | --- | --- | --- | --- | --- | --- | --- |
| 1 | 6-9 | R255X | 13 | yes | 48 mos | no | Trazadone, Depakote | N/A |
| 2 | 6-9 | R133C | 8 | yes | 72 mos | yes | Lovastatin, Topomax, Abilify, Lexapro, Depakene, Nexium | 21 mos |
| 3 | 10-14 | R306C | 7 | yes | 168 mos | yes | Lovastatin, Ambien, Trazodone | 50-60 mos |
| 5 | 10-14 | C964C | 5 | no | no | yes | Trazadone | 156 mos |
| 7 | 6-9 | deletion | 9 | no | no | yes | Lovastatin | 11 mos |
| 8 | 10-14 | R255X | 6 | no | no | yes | Lovastatin | 12 mos |
| 9 | 15-19 | R270X | 14 | N/A | N/A | no | none | N/A |
| 13 | 10-14 | deletion | 14 | yes | 30 mos | no | Depakene, Lexapro,Lactulose | N/A |
| 14 | 10-14 | Q170x mutation at nucleotide c508 | 10 | N/A | N/A | yes | N/A | 18 mos |
| 16 | 10-14 | deletion | 12 | no | 72 mos | yes | Risperdol and Necon | 144 mos |
| 17 | 20-24 | R133C | 12 | yes | 108 mos | yes | Depakote, Carintor, Artane, Copaxone | 17 mos |
| 18 | 10-14 | deletion | 11 | N/A | N/A | no | Depakene | N/A |
| 19 | 15-19 | R270X | 13 | no | N/A | no | N/A | N/A |
| 21 | 15-19 | 418ins4 | 15 | yes | N/A | no | Diastat, Depakene, Carnitor | N/A |
| 22 | 20-24 | T158M | 11 | yes | 72 mos | yes | Lexapro, Lamictal | 15-18 mos |
| 23 | 6-9 | Large deletion | 12 | yes | 60 mos | no | Valproic acid | N/A |
| 24 | 6-9 | Deletion exon 3&4 | 9 | yes | 36 mos | no | Keppra, deplane | N/A |
| 25 | 10-14 | T158M | 12 | no | N/A | no | Nanadol, prevacid | 24 mos |
| 4 | 15-19 | T158M | 11 | yes | 120 mos | yes | Copaxone, Topomax, Lovastatin, | 10 mos |
| 6 | 6-9 | R294X | 7 | no | no | yes | Lexapro, Lovastatin, Prilosec | 17 mos |
| 10 | 10-14 | R306C | 9 | no | no | yes | Lexapro | 18 mos |
| 11 | 15-19 | R306C | 10 | no | no | yes | Lexapro, Copaxone, Trazodone, | N/A |
| 12 | 6-9 | deletion | 7 | yes | 24 mos | no | Depakene, Lactulose, Lovastatin, | 30 mos |
| 15 | 10-14 | T158M | 10 | N/A | N/A | no | N/A | N/A |
| 20 | 6-9 | R133C | 10 | yes | 42 mos | yes | Abilify, Lexapro, Desyrel | 24 mos |

**Table S1.** Clinical demographics of all enrolled participants with Rett syndrome. The shaded area depicts excluded participants. RSSS = Rett Syndrome Severity Score; Seizure OS = Seizure on set; Age of R = Age of regression in months (mos.); N/A = Not Available.

|  | **TD** (N=24)  Mean Age: 12.3 ± 4.7 | | | **RTT** (N=17)  Mean Age: 12.6 ± 4.8 | | |
| --- | --- | --- | --- | --- | --- | --- |
|  | **450 SOA** | **900 SOA** | **1800 SOA** | **450 SOA** | **900 SOA** | **1800 SOA** |
| Average # of Accepted  Trials ± SD | 412.5 ± 153 | 718.5 ± 96 | 720 ± 81 | 239.1 ± 81 | 507.6 ± 153 | 460.2 ± 96 |

**Table S2.** Number of accepted standard trials included in the analysis across conditions in typically developing (TD) controls and in participants with Rett syndrome.

| **TD** (N=24) | | | **RTT** (N=17) | | |  |  |
| --- | --- | --- | --- | --- | --- | --- | --- |
| **450 SOA** | **900 SOA** | **1800 SOA** | **450 SOA** | **900 SOA** | **1800 SOA** |  |  |
| **SNR** | Pre-DSS Mean ±SEM | 21.09 ± 2.77 | 30 ± 3.25 | 26.55 ± 3.4 | -0.81 ± 3.72 | 9.29 ±4.76 | 14.88 ± 2.86 |
|  | Mean ± SEM Across Conditions | 26.13 ± 0.64 | | | 7.75 ± 0.91 | | |
|  | Post-DSS ± SEM | 30.98 ± 3.25 | 49.75 ± 3.86 | 43.46 ± 3.69 | 23.13 ± 3.56 | 30.29 ± 4.26 | 36.76 ± 5.02 |
|  | Mean ± SEM Across Conditions | 41.4 ± 8.45 | | | 30.06 ± 7.29 | | |
| **ITV** | Pre-DSS Mean ± SEM (µv) | 3.57 ± 0.3 | 3.55 ± 0.3 | 3.54 ± 0.29 | 7.35 ± 0.88 | 7.93 ± 1.03 | 8.01 ± 1.05 |
|  | Mean ±SEM (µv) Across Conditions | 3.55 ± 0.29 | | | 7.76 ± 0.97 | | |
|  | Post-DSS ± SEM (µv) | 1.68 ± 0.14 | 1.52 ± 0.13 | 1.41 ± 0.12 | 1.59 ± 0.21 | 1.72 ± 0.23 | 1.95 ± 0.21 |
|  | Mean ±SEM (µv) Across Conditions | 1.54 ± 0.13 | | | 1.75 ± 0.21 | | |

**Table S3.** Mean ± SEM for SNR and ITV, Pre and Post–DSS, for each condition in typically developing (TD) controls and in participants with Rett syndrome (RTT).
